# The spatiotemporal distribution of water quality characteristics of the tropical, transboundary Sio Malaba Malakisi River Basin using multivariate statistical techniques

**DOI:** 10.1007/s10661-025-14282-1

**Published:** 2025-07-05

**Authors:** Hope Mwanake, Moritz Feigl, Bano Mehdi-Schulz, Nzula Kitaka, Karsten Schulz, Luke O. Olang, Jakob Lederer, Mathew Herrnegger

**Affiliations:** 1 Department of Landscape, Water and Infrastructure, Institute of Hydrology and Water Management, BOKU University, Muthgasse 18, 1190 Vienna, Austria; 2 Department of Biological Sciences, Egerton University, P.O. Box 536—20115, Egerton-Njoro, Kenya; 3 Department of Biosystems and Environmental Engineering, Technical University of Kenya, Nairobi, Kenya; 4 Institute of Chemical, Environmental and Bioscience Engineering, TU Wien, Getreidemarkt 9/166, 1060 Vienna, Austria; 5 Institute for Water Quality and Resource Management, TU Wien, Karlsplatz 13/226, 1040 Vienna, Austria; 6 Baseflow AI Solutions GmbH, Burggasse 58/12A, 1070 Vienna, Austria

**Keywords:** Sio Malaba Malakisi catchment, Water quality distribution, Spatiotemporal variation, Multivariate statistical techniques, Transboundary river basin, Anthropogenic activities, And agricultural impact

## Abstract

Surface water pollution driven by land use practices and soil erosion remains a persistent challenge in tropical river basins of East Africa. Despite its socio-economic importance, the transboundary Sio Malaba Malakisi River Basin (SMMRB), shared by Kenya and Uganda, lacks comprehensive data on spatial and seasonal water quality dynamics. This study provides the first year-long baseline assessment of surface water quality in the SMMRB, using water samples collected from 12 monitoring sites across three distinct hydrological seasons: dry, short rainy, and long rainy. Twelve physicochemical parameters were analyzed following standardized protocols from the American Public Health Association (APHA), resulting in 854 data points. Multivariate statistical techniques: agglomerative hierarchical clustering, Wilk’s lambda analysis, and exploratory factor analysis (EFA), were used to identify patterns and key drivers of water quality variation. Three distinct spatial clusters, corresponding to the Sio, Malaba, and Malakisi sub-catchments, were identified, each exhibiting unique water quality profiles. Elevated concentrations of total phosphorus (TP), soluble reactive phosphorus (SRP), and total suspended solids (TSS) were observed, exceeding typical background levels for unpolluted rivers. Seasonal differences highlighted the role of sediment transport and dilution processes, particularly during the rainy seasons. These findings provide novel insights into nutrient transport and hydrogeomorphological influences in a tropical, data-scarce, transboundary basin. The results offer a scientific basis for setting up targeted monitoring stations and adaptive water management strategies. Future studies should assess long-term interactions between sediment and nutrients. Evaluating the effectiveness of soil and water conservation practices will also be important for improving water quality.

## Highlights

- This study presents the first comprehensive assessment of water quality dynamics in the transboundary Sio–Malaba–Malakisi River Basin (SMMRB).
- Multivariate statistical techniques (hierarchical clustering, Wilk’s lambda analysis, and factor analysis) were used to identify key drivers of spatial and seasonal water quality variation.
- Hierarchical clustering revealed distinct spatial patterns aligned with sub-catchments, supporting targeted management approaches.
- Elevated concentrations of total suspended solids (TSS) and phosphorus forms (TP and SRP) were identified as the primary water quality concerns in the basin.
- The findings provide a scientific framework for establishing future water quality monitoring stations and inform adaptive watershed management in tropical transboundary basins.

## Introduction

East African nations such as Kenya, Tanzania, and Uganda face significant challenges due to the limited availability of reliable and freely accessible water quality and quantity data. (Devi & Bostoen, 2009; Scholz et al., 2007; Wynants et al., 2019). This limitation hampers the application of water management tools, including hydrological models and water quality assessments, which are necessary for informed decision-making (Calow et al., 2010; Garibay et al., 2022; Tampo et al., 2024).

The population density in East Africa is considered moderately high, as of 2025, approximately 79 people per square kilometer (World Population Review, 2025), causing increasing pressure on land resources. This leads to the practice of several unsustainable agricultural practices, such as cropping within riparian zones or converting wetlands and forests to agriculture (Bamutaze et al., 2021; Barasa et al., 2011; Kaindi, 2013; Mondorf, 2020). These practices contribute to erosion, nutrient runoff, and alterations in stream flow, primarily affecting downstream human and natural communities (Olago & Odada, 2007). The risk of soil erosion has increased due to the conversion of natural vegetation to agriculture, land fragmentation, and unsustainable farming methods: lack of soil and water conservation measures, and cultivating on steep slopes (Jiang et al., 2014; Mwanake et al., 2023). The water quality of many other inland waters in East Africa, including river ecosystems such as the Athi Galana Sabaki and the Mara rivers (Amann et al., 2021; Dessu et al., 2014; Kamau et al., 2015; Lal, 2003; Montgomery, 2007; Pimentel et al., 1995), has also been harmed by land conversion and erosion processes (Olago & Odada, 2007). During precipitation, excessive sediment and nutrient loads are transported into inland waters as surface runoff, primarily because of deforestation and erosion (Amann et al., 2021; R. Mwanake et al., 2022; Olago & Odada, 2007).

The Sio Malaba Malakisi River Basin (SMMRB), partially draining into Lake Victoria, is a transboundary catchment located along the border region between Kenya and Uganda. Characterized by heterogeneous topography, the basin encompasses flat areas with low soil erosion risk and steep hills prone to severe erosion, particularly towards Mount Elgon (4321 masl) (Mwanake et al., 2023). Comprising two main river catchments (the Sio catchment and the Lwakhakha Malaba Malakisi catchment), the SMMRB spans 78% of its area in Kenya and 22% in Uganda. Over 80% of the basin is agricultural, with various crops cultivated.

High population density and growth in the catchment have resulted in intense land fragmentation, expanding agriculture into erosion-prone riparian zones and wetlands. While water quality challenges are evident in the SMMRB, historical data is scarce, and no comprehensive baseline analysis of its water quality characteristics exists in either Kenya or Uganda. Existing water quality studies in the SMMRB primarily focus on localized areas and employ inconsistent sampling methods (Kaindi, 2013). This fragmented approach hinders the establishment of a reliable understanding of water quality across the Sio, Lwakhakha, Malaba, and Malakisi rivers. There is a clear need for consistent, basin-wide monitoring to facilitate effective analysis and sustainable management of the transboundary water resources.

To address this gap, this study presents a multidisciplinary approach of integrating the first assessment of nutrient pollution and sediment transport across the SMMRB by combining: one year of field sampled water quality data; laboratory analyses; spatial and seasonal clustering; and exploratory factor analysis (EFA) to provide novel insights into the key drivers of nutrient variability to offer actionable recommendations for catchment scale monitoring and management of the basin. The surface waters of the SMMRB serve diverse purposes, including agricultural, commercial, domestic use, and drinking water provision. In this study, we prioritized the physicochemical parameters: dissolved oxygen (DO), conductivity (Cond), water temperature (WT), total suspended solids (TSS), nitrogen, and phosphorus. These parameters are crucial for maintaining drinking water standards and ensuring overall surface water health. Our selection aligns with previous studies that consistently identify issues related to soil erosion (evidenced by high turbidity and color, particularly during rainy periods) and pollution stemming from wastewater, industrial sources, and land use practices.

The study contributes to a better understanding of the basin’s characteristics and water quality dynamics by analyzing the sampled parameter similarities and differences within the sampling sites. As well as how the water quality parameters vary spatially and seasonally throughout the SMMRB. This is important for establishing a baseline understanding of the current state of water quality within the basin as a reference point for future assessments, including (i) decisions on the design of monitoring campaigns, including the identification of sampling sites and parameters to sample, and their sampling frequency ensuring optimized use of resources for field campaigns; (ii) identifying potential “hotspots” of contamination or degradation within the basin to allow targeting mitigation efforts either preventive or remedial measures; (iii) proper resource allocation to areas most in need of intervention to address pollution effectively; (iv) enabling stakeholders and decision makers to develop more effective management strategies and policies; and (v) protecting ecosystem health and human well-being.

The baseline water quality data obtained from the fieldwork serve as a crucial foundation for future studies within the basin to integrate observational insights with ecohydrological models to achieve various objectives, such as assessing sediment yield and water quality dynamics within the basin. By addressing the problem of data unavailability and the innovative strategies undertaken to overcome this challenge, this study contributes to the broader discourse on the complexities of conducting hydrological research in data-deficient environments.

This research addresses major objectives:

1. To conduct a comprehensive assessment of water quality parameters within the Sio Malaba Malakisi River Basin (SMMRB) to establish a baseline understanding of water quality dynamics.
2. To identify the key water quality parameters that drive spatial and seasonal variability across the SMMRB, and to examine how these key parameters differ among the identified spatial clusters.
3. To assess how seasonal changes influence spatial variability in water quality across the SMMRB, and to identify sub-catchments with elevated levels of key pollutants that may serve as potential “hotspots” of contamination within the basin.

## Methods and materials

### Background information on the case study area

**Fig. 1:**
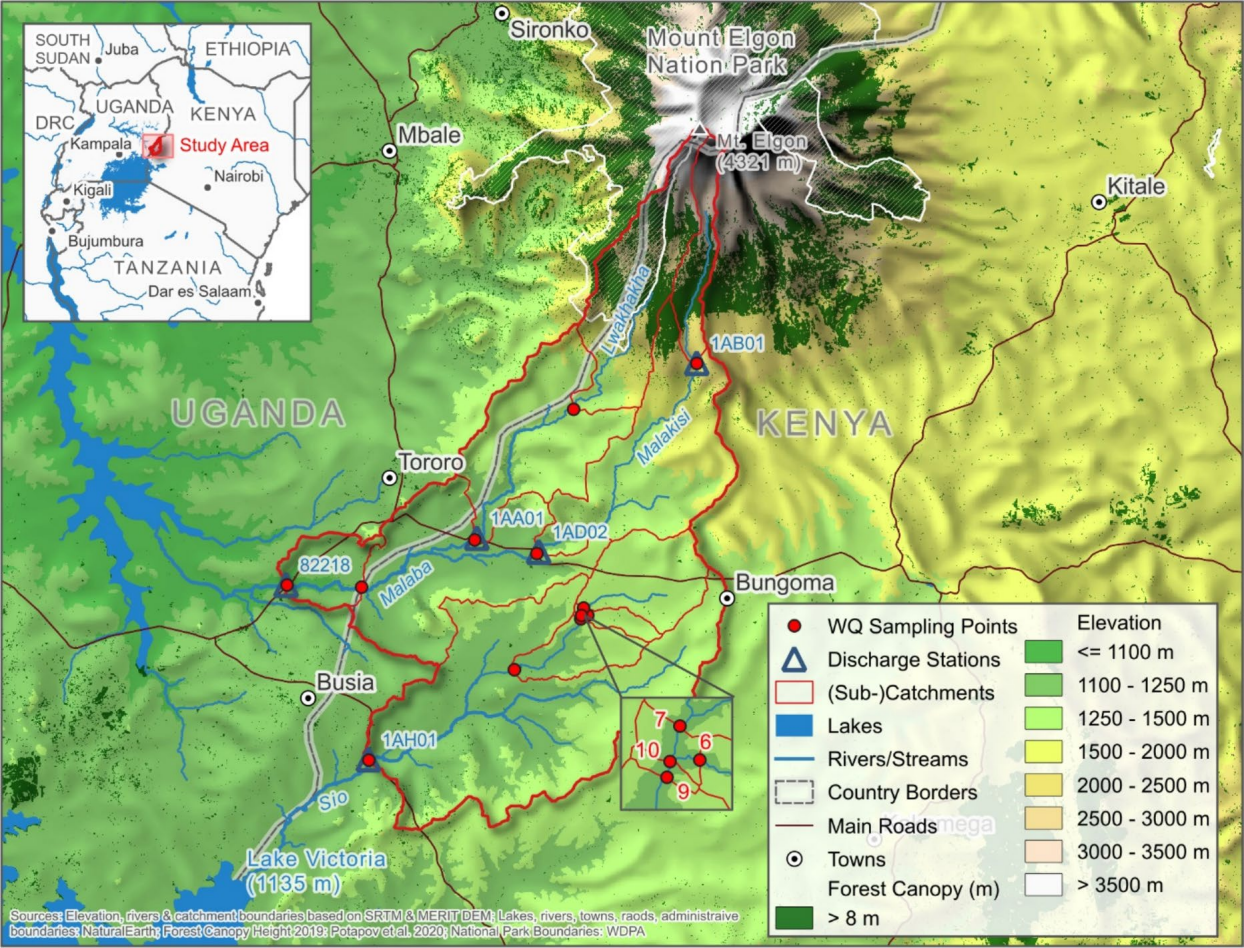
A map showing the Sio Malaba Malakisi River Basin, together with the water quality sample sites indicated by the numbers

The Sio Malaba Malakisi River Basin, situated in the border region between Kenya and Uganda, is delineated by Mount Elgon to the north and Lake Victoria to the south, spanning from latitude 1.133° north to 0.193° south and longitude 33.673° west to 34.571° east (Fig. 1). This region encompasses two primary river catchments, namely the Sio catchment and the Lwakhakha Malaba Malakisi catchment, covering a total area of 3022 km^2^, with approximately 78% of the area located in Kenya and the remaining 22% in Uganda.

According to Kaindi (2013), the SMMRB is characterized by a wide range of soil types exhibiting considerable fertility and variation in drainage properties. The upper slopes of Mt. Elgon have three soil types: andosols (eutrophic soils of tropical regions), nithosols (or ferrisols), and histosols (hydromorphic soils). These soils are volcanic in origin, fertile, and rich in minerals. The soils in the middle lying reaches of the basin are comprised of well-drained, moderately deep to very deep, reddish brown to yellow–brown, friable clay. Along the river valleys, the soils consist of a complex of imperfectly drained to poorly drained soils, often underlying a topsoil of friable sandy clay loam.

Mwanake et al. (2023) have provided a comprehensive description of the catchment area, including details on hydrology, climate, land use, land cover, agriculture, soil erosion, as well as socio-economic, socio-cultural, and political aspects.

This study conducted a comprehensive, year-long, cross-seasonal water quality assessment to address knowledge gaps and facilitate informed decision-making in the SMMRB. Samples were collected from 12 strategic sites along the Sio, Malaba, Malakisi, and Lwakhakha rivers and analyzed. Multivariate statistical analyses are employed to identify potential pollution hotspots, assess differences between seasons, and determine key water quality drivers within the SMMRB. This information will inform future decisions on the design of monitoring campaigns, including the identification of sampling sites and parameters to sample, as well as their sampling frequency. Additionally, it will help pinpoint potential pollution sources, ensuring optimized use of resources for field campaigns and facilitating the design of preventive or remedial measures to address pollution effectively.

The research investigates variations in water chemistry (including nitrogen, phosphorus, sediment levels, dissolved oxygen, and pH) across the SMMRB by season and location.

### Sampling sites and analytical procedure

Twelve sampling sites were chosen to accurately represent the primary course and tributaries of the Sio, Malaba, Malakisi, and Lwakhakha rivers, as illustrated in Table 1 and Fig. 1. These sampling sites are strategically located to encompass the upper, middle, and lower reaches of each river, ensuring comprehensive coverage of the study area’s hydrological dynamics and spatial coverage of key flow paths and confluences. In addition to hydrological representativeness, site selection considered accessibility and proximity to known land use pressures such as agricultural zones and settlements. Where possible, sites were also selected to reflect variation in landscape features, including slope and vegetation cover, which influence runoff and sediment transport.

**Table 1:** Water quality sampling sites with their coordinates, rivers, and catchments represented across the SMMRB

Number allocated from Fig. 1	Water quality site	Site	Catchment	River	Longitude	Latitude
1	Middle Busia Wetland, Uganda	MBWU	Malaba	Malaba	34.138192	0.58314
2	Tororo Jinja Road (82218)	TJ	Malaba	Malaba	34.051911	0.585483
3	Busia–Kisumu Road (1AH01)	KB	Sio	Sio	34.146255	0.383754
4	Bungoma Malaba Bridge/Amagoro (1AD02)	AM	Malakisi	Malakisi	34.34183	0.62294
5	Malaba Kenya Uganda Border	MKUB	Malaba	Malaba	34.268488	0.63809
6	Nandigwa Upstream Myanga	NU	Sio	Nandigwa	34.398149	0.551147
7	R. Sio Point 2	S2	Sio	Sio	34.394051	0.558679
8	R. Sio Point 1	S1	Sio	Sio	34.314479	0.487427
9	After Confluence_Mayanja-Nandingwa Myanga	Ma	Sio	Mayanja	34.391971	0.550199
10	Before Confluence/Nandigwa Myanga	N	Sio	Nandigwa	34.392071	0.550524
11	Lwakhakha	L	Malaba	Lwakhakha	34.380842	0.791241
12	Kamabus Cheptais (1AB01)	KC	Malakisi	Malakisi	34.525617	0.843129

Four of the sampling sites correspond to sampling stations and discharge gauges managed by the Water Resource Management Association (WRMA) in Kenya and the Ugandan Ministry of Water and Environment. These sites are denoted as km (1AB01), KB (1AH01), AM (1AD02), and TJ (82218), with their official designations provided in brackets in Table 1. The selection of sampling points along the upper reaches was guided by accessibility to headwater stream points, with some sites, like KC, situated in the elevated terrains of Mount Elgon, particularly within the upper reaches of the Malakisi River.

**Fig. 2:**
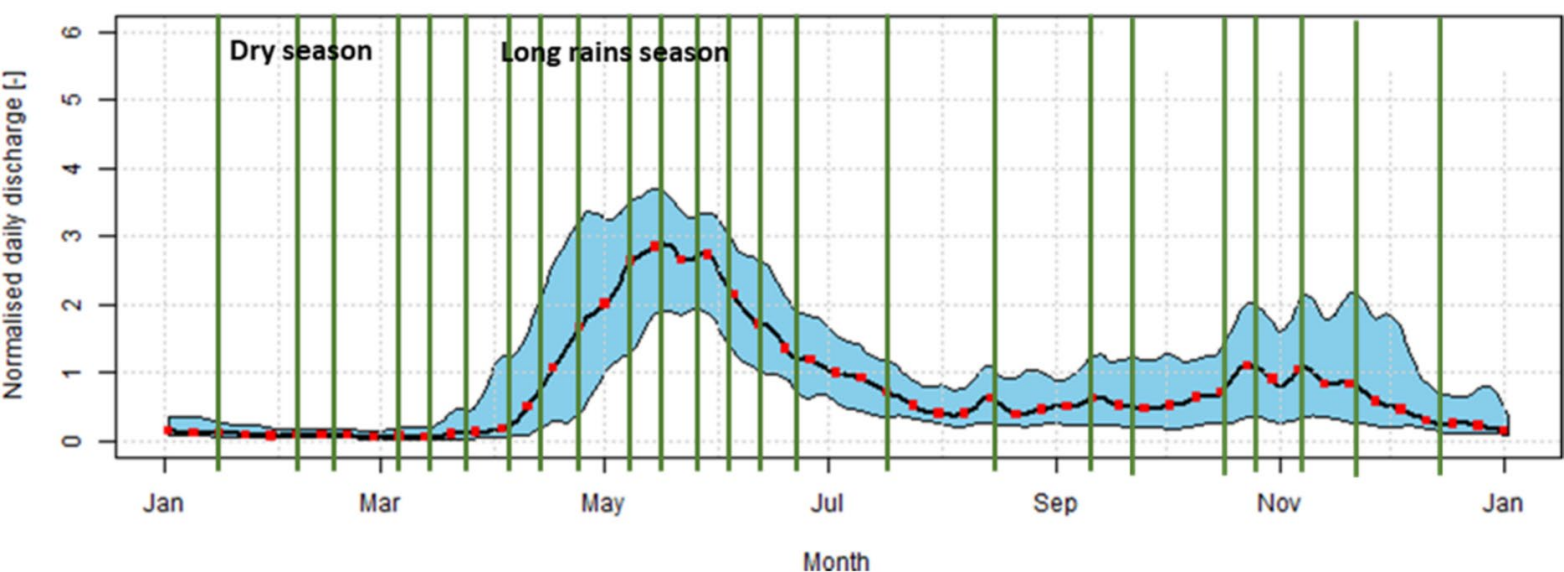
Sampling campaigns (24 in number), including the distribution of historic discharge, illustrated for the Sio River

Additionally, four assessment sites (S2, NU, N, and Ma) along the River Sio were included to monitor changes in water quality at locations where two river tributaries converge. Furthermore, one sampling site (MBWU) is positioned in a wetland area in Uganda to observe its impact on water quality at the subsequent site, TJ. A comprehensive water quality assessment was conducted over one year, from January 23, 2019 to December 21, 2019, as delineated in Table 2 and Fig. 2. In Fig. 2, the green lines illustrate the sampling campaigns 1 to 24 to the historical discharge distribution of the Sio River.

**Table 2:** Table showing the sampling schedule according to the seasons

Campaigns	Date	Season represented
1	1/23/2019	Dry (January)
2	2/5/2019	Dry (February)
3	2/23/2019	Dry (February)
4	3/2/2019	Dry (March)
5	3/9/2019	Dry (March)
6	3/14/2019	Dry (March)
7	3/26/2019	Dry (March)
8	4/2/2019	Dry (April)
9	4/9/2019	Dry (April)
10	4/24/2019	Long rainy (April)
11	5/15/2019	Long rainy (May)
12	5/22/2019	Long rainy (May)
13	5/28/2019	Long rainy (May)
14	6/5/2019	Long rainy (June)
15	6/12/2019	Long rainy (June)
16	6/19/2019	Long rainy (June)
17	7/2/2019	Long rainy (July)
18	8/16/2019	Short rainy (August)
19	9/7/2019	Short rainy (September)
20	9/26/2019	Short rainy (September)
21	10/12/2019	Short rainy (October)
22	10/26/2019	Short rainy (October)
23	11/19/2019	Short rainy (November)
24	12/21/2019	Short rainy (December)

Water samples were systematically collected in triplicate from the midstream section of the river, positioned to face the flow of the river system. Depending on the site condition as well as the season (dry or rainy), samples were taken either from the river banks (at accessible shallow points), by wading into the stream (where safe and feasible), or directly from the bridge using clean, acid-prewashed bottles. At each site, water was sampled approximately 20–30 cm below the surface to avoid surface contaminants, and all sample bottles were thoroughly rinsed with the river water before the final sample collection. See Appendix 1 for how the samples were preserved, transported to the laboratory, and how they were analyzed. A total of 854 samples were gathered and subjected to analysis of various physico-chemical properties, as outlined in Table 3. The samples were processed according to the standard methods outlined by the American Public Health Association (APHA, 2012). Additionally, the discharge (Q) measurements were obtained using the velocity area method, following standard hydrological procedures, and the parameters necessary for calculating discharge were also recorded, comprising the wetted cross-sectional channel area (*A*), velocity (*V*), and depth (*D*). A measuring tape was stretched across the river cross-section to define the width at each site. Depth measurements were taken at regular intervals (this was dependent on the river width) using a calibrated wading rod. At each vertical point, water velocity was measured at 60% of the total depth from the surface as per standard guidelines.

**Table 3:** Water quality parameters were collected and analyzed according to the protocol by APHA, 2012

Type	Parameter	Abbreviation	SI Unit	Analytical procedure
Sediment concentration	Total suspended solids	TSS	mg/L	Gravimetric method
Nitrogen parameters	Total nitrogen	TN	mg/L	Persulfate method (Koroleff, 1983)
	Nitrate-nitrogen	NO_3_^−^ − N	mg/L	the sodium-salicylate method
	Nitrite-nitrogen	NO_2_^−^ − N	μg/L	Griess-Saltzman method
	Ammonium-nitrogen	NH_4_^+^ − N	μg/L	hypochlorite method
Phosphorus parameters	Total phosphorus	TP	μg/L	Persulfate method (Koroleff, 1983), then the ascorbic acid method
	Soluble reactive phosphorus	SRP	μg/L	the ascorbic acid method
Physico-chemical parameters	Water temperature	WT	°C	In situ
	Conductivity	Cond	μS/cm	In situ
	Dissolved oxygen	DO	mg/L	In situ
	% oxygen saturation	%Sat	%	In situ
	pH	pH	Dimensionless	In situ
Discharge parameters	Velocity	V	m/s	Velocity meter
	Chanel area	A	m^2^	Length and width Using a rope and a tape measure
	Depth	D	m	Stick and a measuring tape

Water velocity was measured using the floaters and a rotating element flow meter. The wetted cross-sectional area (representing the portion of the channel actively conveying water at the time of sampling and varied across seasons due to changes in water levels) was calculated by summing the product of each depth and corresponding segment width, and the discharge was determined by multiplying the average velocity by the cross-sectional area. (see Appendix 3).

More information on data handling is given in Appendix 1.

### Statistical methods for data analysis

All programming was conducted in R version 4.03 (R Core Team 2020).

Different statistical methods were employed to examine spatial variations (across river sites), temporal patterns (wet/dry seasons), and similarities in water quality parameters.

For spatial pattern analysis, agglomerative hierarchical clustering was utilized to delineate groups of sites exhibiting similar water quality (WQ) parameters. This approach is chosen over simple correlation analysis because the high-dimensional nature of the dataset requires an analysis that can handle multiple parameters simultaneously, capturing the complex interactions and variability within the dataset. Computing correlation values for individual parameters could be misleading, as they might not fully represent the multi-parameter interactions that influence water quality within the various sub-catchments in the SMMRB catchment.

Temporal patterns were assessed by comparing water quality parameters across the three seasons. Analysis of variance (ANOVA) was conducted to test for differences in water quality parameters among spatial and temporal groups, with Tukey’s HSD multiple comparison procedure used to evaluate pairwise differences.

Wilk’s *λ* quotient distribution was employed to identify parameters playing the most significant role in cluster group formations (Wilks, 1932). This method allows for the identification of key drivers of variability across multiple dimensions, which is crucial for understanding the comprehensive impact of various parameters on water quality.

Agglomerative hierarchical clustering was then utilized to objectively derive groups of the most influential parameters that influence the water quality of the SMMRB (i.e., those with low *λ* values). This step ensures that the analysis considers the most significant factors affecting water quality, providing a more accurate and meaningful interpretation of the data.

Finally, a factor analysis was carried out to evaluate the water quality variation characteristics of the various spatial clusters. Factor analysis helps reduce the dimensionality of the dataset and identify underlying factors that explain the observed variability in water quality parameters across different spatial clusters. The suitability of the data for exploratory factor analysis (EFA) was assessed using the Kaiser–Meyer–Olkin (KMO) measure and Bartlett’s test of sphericity. The overall KMO values (ranging from 0.53 to 0.64) were within the acceptable range for EFA in exploratory environmental studies, and Bartlett’s test was highly significant (*p* < 0.0001), indicating that the data were appropriate for factor extraction.

To mitigate biases stemming from differing water quality parameter scales, all parameters were standardized prior to applying hierarchical clustering and factor analysis. Predictive mean matching (PMM) (Rubin, 1986) was utilized to impute 45 missing values (0.6% of observations) in the variables conductivity, pH min, pH max, dissolved oxygen (DO), and dissolved oxygen saturation (%Sat). PMM exhibited superior model performance measures and yielded the least biased estimates compared to other methods (Marshall et al., 2010). It was also found to perform effectively across a variety of scenarios (Kleinke, 2017). A significance level of 0.05 was set for all statistical tests.

The clustering methods relied on the cluster package (Maechler et al., 2021), factor analysis was performed using psych (Revelle, 2021), and visualizations were generated using ggplot2 (Wickham, 2016). More details on the statistical methods are given in Appendix 2.

## Results and discussions

### Baseline water quality assessment

The descriptive statistics of the measured WQ parameters for three rivers in their sub-catchments for 1 year (2019) are presented in Table 4.

**Table 4:** Descriptive statistics of the water quality (WQ) parameters in 2019 of the SMMRB rivers (*n* = 854)

Parameter	Abbreviation	Units	Minimum	Maximum	Mean	Standard deviation	Minimum	Maximum	Mean	Standard deviation	Minimum	Maximum	Mean	Standard deviation
Dry season subcatchments														
Water temperature	WT	°C	21.6	31.2	**26.7**	2.4	20.7	26.7	23.5	1.4	17.4	29.9	23.1	4.6
Conductivity	Cond	μS/cm	74.5	361.0	202.6	69.6	74.9	455.7	222.1	96.4	68.1	374.7	**225.9**	84.3
Dissolved oxygen	DO	mg/L	5.75	8.26	6.91	0.64	0.97	6.60	**4.23**	1.52	6.45	8.78	**7.42**	0.60
Percentage oxygen saturation	%Sat	%	86.23	114.20	96.92	6.35	13.17	91.87	**58.19**	22.56	91.53	128.27	103.43	9.94
Minimum pH	pHmin	unit less	6.8	9.8	–	–	6.2	9.0	–	–	6.5	9.00	-	-
Maximum pH	pHmax	unit less	7.0	**10.7**	–	–	6.5	9.8	–	–	6.5	10.4	-	-
Total suspended solids	TSS	mg/L	8.33	604.04	**117.82**	114.72	8.33	222.22	42.50	42.48	10.00	228.89	70.06	69.76
Total phosphorus	TP	μg/L	37.14	328.57	109.56	67.32	37.14	328.57	109.56	67.32	61.90	521.90	**221.34**	144.55
Soluble reactive phosphorus	SRP	μg/L	12.86	130.48	**71.15**	33.18	0.00	103.81	13.19	17.97	0.00	131.90	65.61	37.12
Total nitrogen	TN	mg/L	0.34	2.78	1.11	0.75	0.37	2.73	**1.20**	0.74	0.33	2.63	0.92	0.65
Nitrate–nitrogen	NO_3_^−^ − N	mg/L	0.00	1.23	0.38	0.28	0.00	0.57	0.14	0.12	0.06	0.75	**0.38**	0.23
Nitrite–nitrogen	NO_2_^−^ − N	μg/L	1.71	100.19	18.00	19.81	0.00	81.05	12.10	15.83	3.52	57.24	**18.10**	17.64
Ammonium–nitrogen	NH_4_^+^ − N	μg/L	0.00	313.67	41.38	52.06	3.47	1065.13	**132.28**	171.51	0.00	295.00	47.69	65.10
Velocity	V	m/s	0.06	0.65	0.31	0.14	0.00	0.48	0.11	0.12	0.03	0.71	**0.31**	0.20
Discharge	Q	m^3^/s	0.09	1.60	**0.52**	0.36	0.00	1.23	0.07	0.21	0.00	0.76	0.17	0.25
Depth	D	M	0.16	0.48	**0.30**	0.09	0.00	0.66	0.12	0.18	0.00	0.35	0.14	0.12
Wetted channel area (used to calculate discharge)	A	m^2^	0.32	2.83	**1.26**	0.63	0.00	4.54	0.71	1.20	0.00	4.02	1.11	1.45
Short rain season subcatchments														
Water temperature	WT	°C	18.5	28.6	22.9	2.5	21.3	27.8	**22.9**	1.4	16.9	23.9	20.8	2.5
Conductivity	Cond	μS/cm	73.4	150.1	112.3	21.1	80.6	163.7	121.6	19.7	110.3	166.3	**139.9**	17.3
Dissolved oxygen	DO	mg/L	4.03	7.01	6.00	0.89	5.29	6.99	**5.98**	0.47	6.49	6.80	**6.59**	0.11
Percentage oxygen saturation	%Sat	%	58.13	89.63	**80.83**	8.92	69.07	91.77	82.10	6.04	85.57	92.13	88.96	2.43
Minimum pH	pHmin	unit less	7.3	11.6	–	–	6.8	**13.4**	–	–	7.4	13.0	-	-
Maximum pH	pHmax	unit less	7.4	11.8	–	–	6.8	**13.4**	–	–	7.4	13.2	-	-
Total suspended solids	TSS	mg/L	36.11	990.48	**337.84**	224.83	27.78	563.33	163.87	118.80	60.00	520.00	281.06	148.74
Total phosphorus	TP	μg/L	213.81	751.43	**456.15**	137.38	13.81	509.52	189.14	117.68	103.81	563.81	396.34	149.59
Soluble reactive phosphorus	SRP	μg/L	50.95	116.67	**77.28**	16.49	3.81	93.33	40.11	22.96	26.67	107.62	68.16	21.75
Total nitrogen	TN	mg/L	0.11	1.48	0.73	0.40	0.06	1.75	0.77	0.52	0.19	1.80	**0.91**	0.46
Nitrate–nitrogen	NO_3_^−^ − N	mg/L	0.12	1.47	0.49	0.38	0.04	1.65	0.68	0.36	0.21	2.38	**1.08**	0.75
Nitrite–nitrogen	NO_2_^−^ − N	μg/L	2.67	39.90	11.89	8.88	6.76	51.52	**22.69**	11.65	9.71	23.43	13.69	3.72
Ammonium–nitrogen	NH_4_^+^ − N	μg/L	5.47	264.80	96.44	81.74	30.80	358.47	**127.03**	100.53	17.80	228.13	91.13	71.21
Velocity	V	m/s	0.35	2.09	1.11	0.52	0.04	1.26	0.47	0.33	0.37	8.20	**1.42**	1.97
Discharge	Q	m^3^/s	6.19	99.60	**27.59**	26.48	0.05	48.16	5.86	11.15	0.04	22.29	6.60	8.18
Depth	D	M	0.91	9.79	**3.23**	3.46	0.13	3.39	1.02	0.93	0.03	1.26	0.52	0.47
Wetted Channel area (used to calculate discharge)	A	m^2^	10.13	163.16	**47.75**	61.16	0.73	72.55	12.18	18.23	0.01	9.83	3.72	4.18
Long rain season subcatchments														
Water temperature	WT	°C	19.6	30.7	**24.5**	2.7	20.7	27.6	23.1	1.4	17.2	26.9	22.0	3.2
Conductivity	Cond	μS/cm	96.1	181.9	140.0	24.3	56.6	154.6	102.2	28.0	71.1	203.7	**151.1**	38.6
Dissolved oxygen	DO	mg/L	3.14	7.11	**5.84**	0.96	4.66	7.16	6.19	0.64	5.73	6.95	**6.46**	0.41
Percentage oxygen saturation	%Sat	%	48.33	92.63	**82.25**	11.30	61.10	95.13	84.00	8.80	86.93	93.00	89.49	2.33
Minimum pH	pHmin	unit less	0.00	11.08	–	–	0.00	10.48	–	–	7.09	**11.66**	-	-
Maximum pH	pHmax	unit less	7.4	11.2	–	–	6.8	**13**	–	–	7.3	11.7	-	-
Total suspended solids	TSS	mg/L	56.67	1796.67	562.57	435.73	26.67	1006.67	264.80	243.33	37.78	2960.00	**724.17**	886.88
Total phosphorus	TP	μg/L	212.86	3440.95	**904.15**	702.59	61.90	907.62	405.38	230.19	180.00	2900.48	893.12	636.79
Soluble reactive phosphorus	SRP	μg/L	38.10	331.43	126.91	62.61	10.00	232.86	58.29	36.08	50.00	402.38	**139.38**	111.11
Total nitrogen	TN	mg/L	0.88	2.62	1.53	0.39	0.80	2.64	1.48	0.41	1.16	3.05	**1.75**	0.57
Nitrate–nitrogen	NO_3_^−^ − N	mg/L	0.13	2.54	0.83	0.42	0.00	2.77	0.96	0.51	0.12	**5.69**	1.30	1.27
Nitrite–nitrogen	NO_2_^−^ − N	μg/L	6.67	92.57	34.06	20.38	8.00	100.19	35.48	19.14	10.48	134.10	**44.64**	37.04
Ammonium–nitrogen	NH_4_^+^ − N	μg/L	6.80	450.47	89.09	92.88	17.13	567.47	98.99	98.35	6.13	990.80	**151.32**	247.67
Velocity	V	m/s	0.29	1.25	0.74	0.31	0.04	0.98	0.31	0.23	0.31	1.42	**0.77**	0.30
Discharge	Q	m^3^/s	0.97	72.32	**19.12**	21.32	0.00	35.23	4.18	8.79	0.01	5.32	1.56	1.79
Depth	D	M	0.35	9.74	**2.97**	3.49	0.06	3.80	0.72	0.82	0.01	0.41	0.22	0.16
wetted channel area (used to calculate discharge)	A	m^2^	2.41	545.40	**68.38**	135.31	0.03	86.35	10.13	19.54	0.01	6.32	2.05	2.29

Discharge (*Q*) and stream velocity (*V*) were critical in understanding the dynamics of the streamflow. The stream velocity ranged from 0.31 to 1.42 m/s, with the highest discharge recorded (99 m^3^/s) in the Malaba sub-catchment during the short rainy season. This is a result of short, intense rainfall events, causing rapid surges in discharge that cause short-term altered river flow dynamics, thereby affecting water quality. The precipitation during the three seasons reflected the mean discharge measured and showed that the overall hydrological regime of the larger Malaba sub-catchment is influenced by the upper reach sub-catchments. Understanding temporal *Q* fluctuations is crucial for predicting and mitigating the impacts of extreme rainfall events and their impacts on water quality pollution to ensure sustainable water resource management within the larger SMMRB (Hoang et al., 2016; Loizeau & Dominik, 2000; Mueller & Pitlick, 2005; Pedinotti et al., 2012).

The water temperature (WT) measured had a wide range, fluctuating between a minimum of 16.9 °C in the Malakisi sub-catchment and a maximum of 31.2 °C in the Malaba sub-catchment. The mean WT also varied across the three seasons, 26.7 ± 2.4 °C in Malaba, 22.9 ± 1.4 °C in Sio, and 24.5 ± 2.7 °C in Malaba for the dry, short rain, and long rain seasons, respectively. These variations correspond to seasonal influences, such as solar radiation, air temperature, and water levels. Warmer temperatures, typically associated with the dry season, coincide with increased solar radiation, whereas cooler temperatures prevail during the wet rainy season. Observations at sites like KC and N, situated upstream along the Malakisi and Sio rivers, respectively, had lower WT values due to shading from the dense vegetation growing on the riparian zone. Conversely, elevated water temperature observed at, MKUB, AM, TJ, MBWU, KB, and occasionally L, may have been attributed to observed human activities upstream such as bathing, washing, open defecation, and direct waste water discharge near settlements and market centers, as well as reduced riparian shading due to vegetation clearance. Additional activities, including industrial discharge and urban runoff, can contribute to localized increases in WT (Bhatia et al., 2018; Dudgeon et al., 2006; Phiri et al., 2005). WT variations within the SMMRB can influence the metabolic rates of aquatic organisms, aquatic plant growth, and dissolved oxygen levels. Monitoring WT is crucial for assessing environmental health, identifying stressors within the SMMRB, and implementing effective management strategies to safeguard water quality and ecosystem integrity (Brock et al., 2003; Pomati et al., 2011; Shade et al., 2011).

The pH also showed much variation. The minimum (6.2) and maximum (13.4) pH were both recorded in the Sio sub-catchment during the dry and short rainy season, respectively. Overall, pH ranges within the SMMRB (Table 4) indicate diverse chemical compositions and environmental conditions. The pH fluctuations within the SMMRB may result from natural factors, such as the presence of organic matter, as well as anthropogenic factors, such as agricultural runoff, urban pollution, and industrial discharge. Monitoring the pH is important for identifying pollution sources, and implementing remediation measures to protect aquatic ecosystems as well as safeguard water use for drinking and recreation (Farhan et al., 2023).

The water conductivity (Cond) values varied between 68.1 and 374.7 μS/cm in the Malakisi sub-catchment, 74.5–361.0 μS/cm in the Malaba sub-catchment, and 74.9–455.7 μS/cm in the Sio sub-catchment during the dry season, indicating high variability in dissolved ion concentrations during the dry season compared to the rainy season. Elevated conductivity levels often suggest increased anthropogenic influence, particularly in areas affected by runoff from agricultural fields or discharges from settlements. All three sub-catchments are characterized by urban areas, agricultural activities, and natural environments with diverse land use patterns that reflect the high variability in conductivity. Measuring conductivity provides valuable insights into water quality and pollution sources because conductivity acts as a proxy for several substances, e.g., salinity, and can indicate the presence of inorganic fertilizers or sewage. Therefore, it serves as a useful parameter for detecting both point and non-point source pollution in river systems (Jia et al., 2017; David Mondorf, 2021).

Dissolved oxygen (DO) values ranged from 0.97 mg/L in the Sio sub-catchment to 8.78 mg/L in the Malakisi sub-catchment, with both values measured during the dry season. The Malakisi sub-catchment had the highest mean DO values of 7.42 ± 0.60 mg/L, 6.59 ± 0.11 mg/L, and 6.45 ± 0.41 mg/L during the dry, short rain, and long rain seasons, respectively, compared to the Sio and Malaba sub-catchments. These results indicate that the Malakisi waters are characterized by relatively well-oxygenated conditions of > 6 mg/L. This threshold is important because it is widely recognized by both the WHO and the EU Water Framework Directive as a minimum level required to sustain healthy aquatic ecosystems. DO levels above 6 mg/L generally support most aerobic aquatic organisms, including fish and macroinvertebrates, indicating good ecological status. The high DO levels in the Malakisi sub-catchment are due to its headwater location, lower anthropogenic pressures, and the presence of riffle zones that promote aeration. Lower DO levels were recorded in Sio, 4.23 and 5.98 mg/L, and in Malaba, 5.84 mg/L, during the dry, short, and long rain seasons, respectively. Low DO levels are caused by factors such as high water temperatures and organic matter decomposition. However, the biological oxygen demand (BOD) and the chemical oxygen demand (COD) were not measured in this study. These are commonly associated with reduced DO and can lead to anoxic or hypoxic conditions that can harm aquatic life. The decrease in DO levels during the rainy seasons, especially in the Malaba and Sio sub-catchments, suggests increased input of organic matter and nutrient loads from agricultural and surface runoff. These effects may be compounded by higher water temperatures during the rainy seasons (and especially during the short rainy season), which further reduce oxygen solubility and increase the rate of microbial decomposition, contributing to lower DO concentrations. Continuous monitoring and assessment of the DO levels are important for understanding water quality dynamics, identifying stressors, and implementing measures to maintain or improve the overall aquatic health (Farhan et al., 2023).

The percent oxygen saturation (%Sat) ranged from a minimum of 13% in Sio to a maximum of 128% in Malakisi during the dry season, with the highest mean values of 96.92 ± 6.35% in Malaba and 103.43 ± 9.94% in Malakisi during the dry season, reflecting the influence of supersaturated gases. This supersaturation is likely due to biological activity, specifically high levels of photosynthetic activity from aquatic plants and algae observed during fieldwork, which can produce more oxygen than the water can hold under normal conditions. Additionally, physical processes, such as rapid temperature changes or water turbulence, as noted in Malakisi, contribute to the dissolution of oxygen, leading to supersaturated conditions within the water bodies (Marchina et al., 2017). As the Malakisi is the headwater of the SMMRB, both biological (presence of algae that produce oxygen through photosynthesis during the warmer season) and physical processes (turbulence) were observed, supporting the occurrence of supersaturation.

**Fig. 3:**
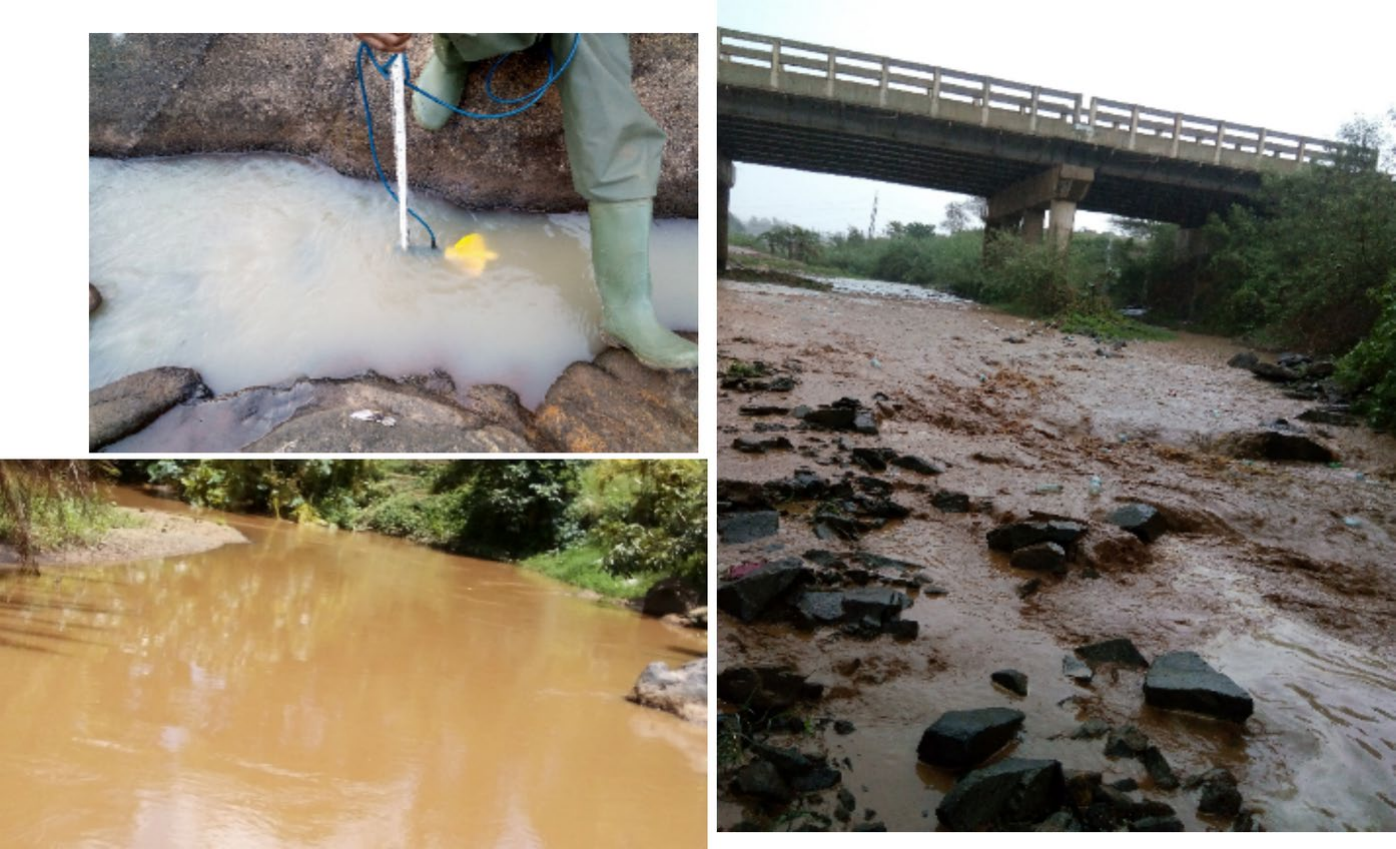
Pictures showing sediment transport by the rivers of the SMMRB taken during the sampling

The rivers of the SMMRB were characterized by turbid waters, as illustrated in Fig. 3, with water quality analysis exhibiting TSS values ranging from 8 mg/L in Malaba during the dry season to 2960 mg/L in Malakisi during the long rain season. The TSS values are markedly higher during the rainy seasons, particularly in the Malakisi sub-catchment. This implies that agricultural regions have considerable runoff with soil erosion, which increases the suspended sediments in the rivers. The water quality could be further deteriorated by contaminants and nutrients (e.g., phosphorus) being carried by the increasing suspended sediments.

The TN values within the SMMRB were low, and the overall concentrations remained well under 5 mg/L; concentrations of TN ranged from 0.06 mg/L in Sio during the short rain season to 3.05 mg/L in Malakisi during the long rain season. The dominant form of inorganic nitrogen is NO_3_^−^ − N, and in high quantities, poses a risk for eutrophication and drinking water. The measured NO_3_^−^ − N values were generally < 1 mg/L and fell within background values due to natural causes (Davies, 1996). The NO_3_^−^ ion is a soluble form of nitrogen and is easily transported by surface and groundwater. The highest NO_3_^−^ − N value was measured in the Malakisi catchment of 5.69 mg/L during the rainy season, which is still well below the WHO maximum permissible levels for drinking water of 10 mg/L (WASREB, 2006; World Health Organization, 2022). In East Africa, low NO_3_^−^ − N values are generally found in surface waters (Esitsakha et al., 2024; Njuguna et al., 2021; Nowicki et al., 2023). The highest NH_4_^+^ − N value was measured in the Sio sub catchment during the dry (1065.13 µg/L) and the short rainy seasons (358.47 µg/L). High NH_4_^+^ − N values may indicate fecal pollution, but since ammonium is not a direct threat to human health, no limit is set by the World Health Organization (WHO). The NO_2_^−^ − N levels measured are < 0.1 mg/L and thus, well below the limit set by WHO of 3 mg/L.

Phosphorus is less soluble than nitrate overall and is mostly transported as soluble reactive phosphorus (SRP), or dissolved inorganic phosphorus, via surface runoff. Phosphorus can also be transported as particulate phosphate attached to soil particles during erosion. TP does not pose a direct threat to human health, but it can cause eutrophication in surface waters, and elevated TP can also be an indication of fecal contamination. Natural background concentrations of TP are < 30 µg/L. The TP concentrations ranged from 37.14 µg/L during the dry season to 3440 µg/L during the long rain season in the Malaba sub-catchment. The SRP levels increase after rainfall events and increases in discharge. For unpolluted rivers, the SRP limit is around 2–20 µg/L (Meybeck, 1982). In the long rainy season, in all three sub-catchments, high mean SRP levels were measured, with the highest mean SRP of 331 ± 127 µg/L measured in the Malaba sub-catchment. Elevated levels of SRP can suggest human impacts resulting from point source pollution, e.g., wastewater discharge, washing clothes, or slaughterhouses. The TP results indicate the influence of both natural processes (weathering, shading, riparian vegetation) and human activities (soil erosion, untreated wastewater) on water quality. These findings show the complexity of the water quality dynamics within the SMMRB and highlight the necessity for consistent, comprehensive monitoring. This is particularly important given that the TP and SRP exceed typical values found in unpolluted rivers worldwide, which range from 10 to 100 µg/L for TP and 5 to 50 µg/L for SRP (Biggs & Close, 1989; Dodds, 2000; Meybeck, 1982).

### Spatial clustering of water quality parameters

Agglomerative hierarchical clustering was used to spatially group the river sampling sites based on their WQ similarities and differences within the SMMRB. Additional WQ parameters, including inorganic and organic total suspended solids, particulate phosphorus, and organic and inorganic nitrogen, were examined for statistical analysis. The clustering analysis revealed distinct patterns among the sampling sites, indicating variations in WQ characteristics across different locations within the basin (Table 4).

**Fig. 4:**
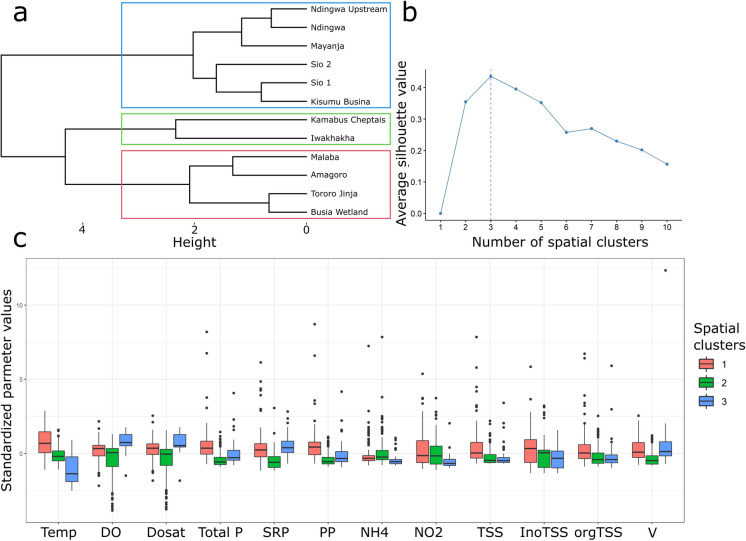
Results of the analysis of spatial similarities in water quality parameters. **a** The dendrogram shows hierarchical clustering results for the SMMRB sites. **b** The average silhouette width for each number of clusters, with the selected number of clusters shown with a dashed line. **c** Boxplots of standardized values of water quality parameters that show significant differences between clusters

**Fig. 5:**
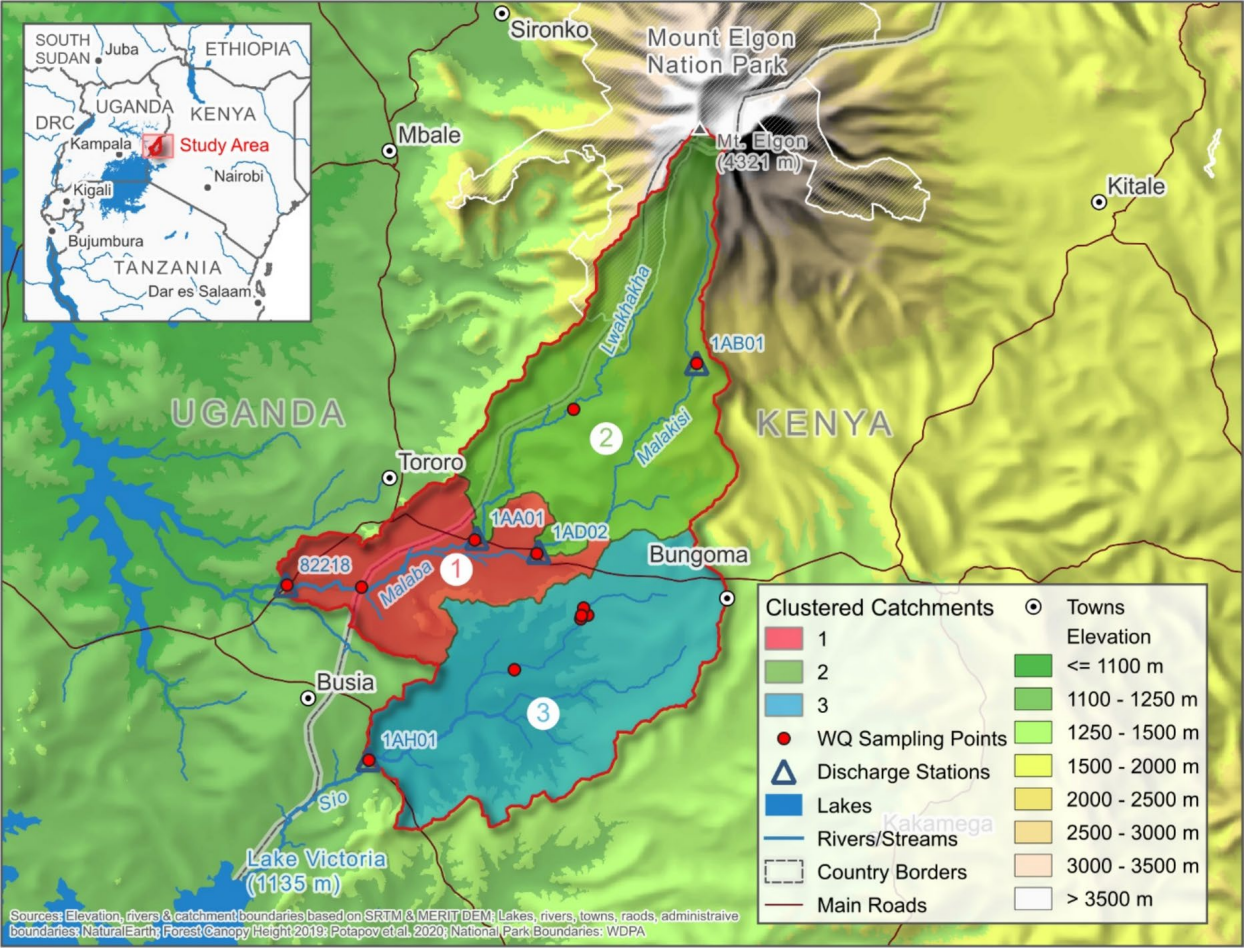
Spatial clusters of water quality sampling sites within the Sio Malakisi Malaba River Basin (SMMRB) were classified based on hierarchical clustering of water quality parameters. Cluster 1 includes sites in the Malaba sub-catchment, with moderate nutrient and sediment levels. Cluster 2 corresponds to the Malakisi sub-catchment, showing comparatively lower nutrient concentrations and representing less anthropogenic influence. Cluster 3 represents sites predominantly in the Sio sub-catchment, characterized by elevated phosphorus concentrations and higher sediment loads. This spatial grouping illustrates the heterogeneity of water quality across the basin and highlights sub-catchment-specific water quality profiles critical for targeted management interventions

The dendrogram generated from the clustering analysis in Fig. 4 shows three different parts: part (a) represents the dendrogram of hierarchical clustering and shows how the SMMRB sites are grouped based on the similarity of WQ parameters. Each branch represents a site, and the length of the horizontal lines represents the distance or dissimilarity between clusters. Sites closely linked at a lower height are more similar to each other; part (b) represents the average silhouette width for the number of clusters. The silhouette width measures how similar an object is to its cluster (cohesion) compared to other clusters (separation). The peak in the silhouette width suggests the optimal number of clusters that best fit the data, which is three for the SMMRB, and part (c) shows the boxplots of standardized WQ parameters for all three spatial clusters of all variables that exhibit significant differences. The dendrogram visually depicted three distinct groups of sampling sites based on their similarity in WQ parameters within the SMMRB. These three groups/clusters corresponded to the three basins, namely the Sio, Malaba, and Malakisi (see Fig. 5), that exhibited each of these basins shared similar WQ profiles, suggesting shared environmental conditions or sources of pollution.

The sites included in each cluster (Fig. 5) include: cluster 1 included NU, N, Ma, S2, S1, and KB, which represent the Sio sub-catchment. Cluster 2 includes KC and L, representing the Malakisi subcatchment, while cluster 3 includes MKUB, AM, TJ, and MBWU from the Malaba subcatchment. The silhouette value (Fig. 4b), which measures the cohesion within clusters and separation between clusters, was calculated to determine the optimal number of clusters. The analysis showed that the sampling sites within the SMMRB were distinct, with clear delineations between clusters based on their WQ attributes. This finding confirms the expected distinction between the sub-basins; however, it is not surprising. What is significant is that we now have objective, derived values to describe spatial variability, reducing subjectivity and potential bias that could arise from a purely qualitative assessment.

From the boxplots (Fig. 4c), it appears the parameters such as WT, DO, %Sat, TP, SRP, NH_4_^+^ − N, NO_2_^−^ − N, V, and TSS, particularly the inorganic TSS, vary across the clusters, which could be indicative of different sources of pollution or natural variations in water composition. These differences need to be taken into account when managing water resources and when implementing soil and water management practices (Khanday et al., 2021; Shrestha & Kazama, 2007). This spatial grouping of the SMMRB into distinct clusters highlights the spatial heterogeneity of WQ within the basin. It highlights the importance of site-specific monitoring and management strategies to address localized environmental challenges.

Table 5 presents the statistical data for various WQ parameters across three different clusters. These clusters represent different groupings based on the similarity of WQ characteristics within the SMMRB. Cluster 1 Malaba, cluster 2 Malakisi, and cluster 3 Sio sub-catchments. Each row of Table 5 corresponds to a specific parameter and provides the mean and standard deviation (SD) of each parameter within each cluster.

**Table 5:** Clusters of sampling sites showing mean values with standard deviations (SD)

	Cluster 1		Cluster 2		Cluster 3	
Parameter	Mean	SD	Mean	SD	Mean	SD
WT (°C)	25.52 (a)	2.51	23.22 (a)	1.41	20.65 (a)	2.67
Cond (μS/cm)	164.22 (c)	67.86	154.31 (c)	83.21	157.09 (c)	54.53
pHmix	8.10 (c)	0.99	7.86 (c)	1.16	8.28 (c)	1.15
pHmax	8.22 (c)	1.03	8.03 (c)	1.26	8.39 (c)	1.2
DO (mg/L)	6.20 (a)	0.88	5.47 (a)	1.44	6.99 (a)	0.71
%Sat (%)	87.14 (a)	13.01	74.00 (a)	19.62	94.59 (a)	10.97
TP (µg/L)	584.83 (a)	482.07	206.55 (a)	184.69	375.68 (a)	327.54
SRP (µg/L)	90.52 (b)	67.35	36.71 (a)	32.71	92.95 (b)	38.04
PP (µg/L)	0.49 (a)	0.43	0.17 (b)	0.16	0.28 (b)	0.3
TN (mg/L)	1.16 (c)	0.67	1.14 (c)	0.61	1.16 (c)	0.62
NH_4_^—^N (µg/L)	98.92 (b)	127.53	119.34 (b)	131.65	43.75 (a)	51.36
NO_3_^—^N (mg/L)	0.66 (c)	0.43	0.58 (c)	0.5	0.70 (c)	0.94
NO_2_^—^N (µg/L)	28.51 (b)	24.84	23.36 (b)	18.71	11.78 (a)	9.93
TSS (mg/L)	421.03 (a)	475.91	154.54 (b)	182.77	168.06 (b)	268.88
V (m/s)	0.68 (b)	0.47	0.28 (a)	0.28	0.84 (b)	1.16

Different letters indicate statistical difference at *p* < 0.05 among clusters with Tukey’s HSD test, with *a* sign. difference to two other clusters; *b*, sign. difference from one another cluster; *c*, no sign. difference from other clusters.

Apart from showing the Table 5 mean and standard deviation (SD) values for all WQ parameters in each cluster, Table 5 also shows an indication of whether these values are significantly different from the other clusters or not, as also seen from Khanday et al.. From the Table 5 results, the parameters that are significantly different across the THREE identified clusters include WT, DO, %Sat, and TP. Most of the parameters are significantly different across the TWO clusters and they include SRP, PP, NH_4_^+^ − N, NO_2_^−^ − N, TSS, and V. These parameters, together with the parameters that are significantly different across the three clusters, can explain the natural, anthropogenic, and seasonal influence of the SMMRB. Specifically, parameters that are significantly different across two clusters, i.e., cluster 1 (Sio) and cluster 2 (Malakisi), include SRP, TSS, and V. The concentrations of SRP and TSS increased from cluster 2 to cluster 1. The V was higher in Malakisi compared to Sio. The clustering analysis revealed distinct differences in water quality parameters across the sub-catchments, reflecting varying environmental conditions and potential sources of pollution. In particular, SRP, NH_4_^+^ − N, NO_2_^−^ − N, and V were significantly different between the Malakisi (cluster 2) and Malaba (cluster 3) sub-catchments. The higher concentrations of SRP in cluster 3 suggest that the Malaba sub-catchment may be more impacted by agricultural runoff or other phosphorus sources compared to the Malakisi sub-catchment. Conversely, higher concentrations of NH_4_^+^ − N, NO_2_^−^ − N, and V in cluster 2 indicate that the Malakisi sub-catchment experiences greater influence from processes such as nitrification and streamflow dynamics. Similarly, differences between the Sio (cluster 1) and Malaba (cluster 3) sub-catchments were marked by higher concentrations of NH4^+^ − N, NO_2_^−^ − N, and TSS in the Sio sub-catchment. This suggests that the Sio sub-catchment may be more prone to erosion and associated particulate phosphorus transport, potentially due to steeper terrain or less effective soil and water management practices. Additionally, the considerably higher conductivity (Cond) in cluster 1 (164.22 ± 67.86 µS/cm) compared to clusters 2 and 3 suggests that the Sio sub-catchment may be more affected by pollution sources, such as urban runoff or industrial discharges. These differences in water quality parameters across the clusters highlight the varying degrees of human and natural influences within each sub-catchment. The Sio sub-catchment, with its higher pollutant concentrations and conductivity, appears to be the most impacted, possibly due to a combination of natural erosion processes and anthropogenic inputs. In contrast, the Malakisi and Malaba sub-catchments, while also affected, display different pollution profiles, likely reflecting the unique land use and hydrological characteristics of each area. The TP has been identified as exceeding natural background values, especially in clusters 1 (Sio) and 3 (Malaba). Ondoo et al. (2019) noted that excessive turbidity and phosphate levels in the Sio sub-catchment were linked to poor farming practices that promoted erosion. It was observed during the field work that the Sio and the Malaba were characterized by numerous housing units with human activities, e.g., bathing and cleaning taking place along the river banks. The spatial distribution of TP, with concentration peaks in these regions, suggests that they are the primary sources of phosphorus pollution within the SMMRB. Both sub-catchments are rapidly growing centers characterized by limited sewage treatment infrastructure and although this study did not directly measure industrial effluents of specific point source discharges, the proximity to unplanned residential developments, informal activities, such as mining and repair workshops for motorbikes could indicate that both domestic and light industry waste water may be a contributing factor to the elevated pollution loads. This observation shows the importance of integrating land use planning and water quality management, as well as the need to map and quantify point and non-point sources within the SMMRB.

### Identifying drivers of spatial variability

**Fig. 6:**
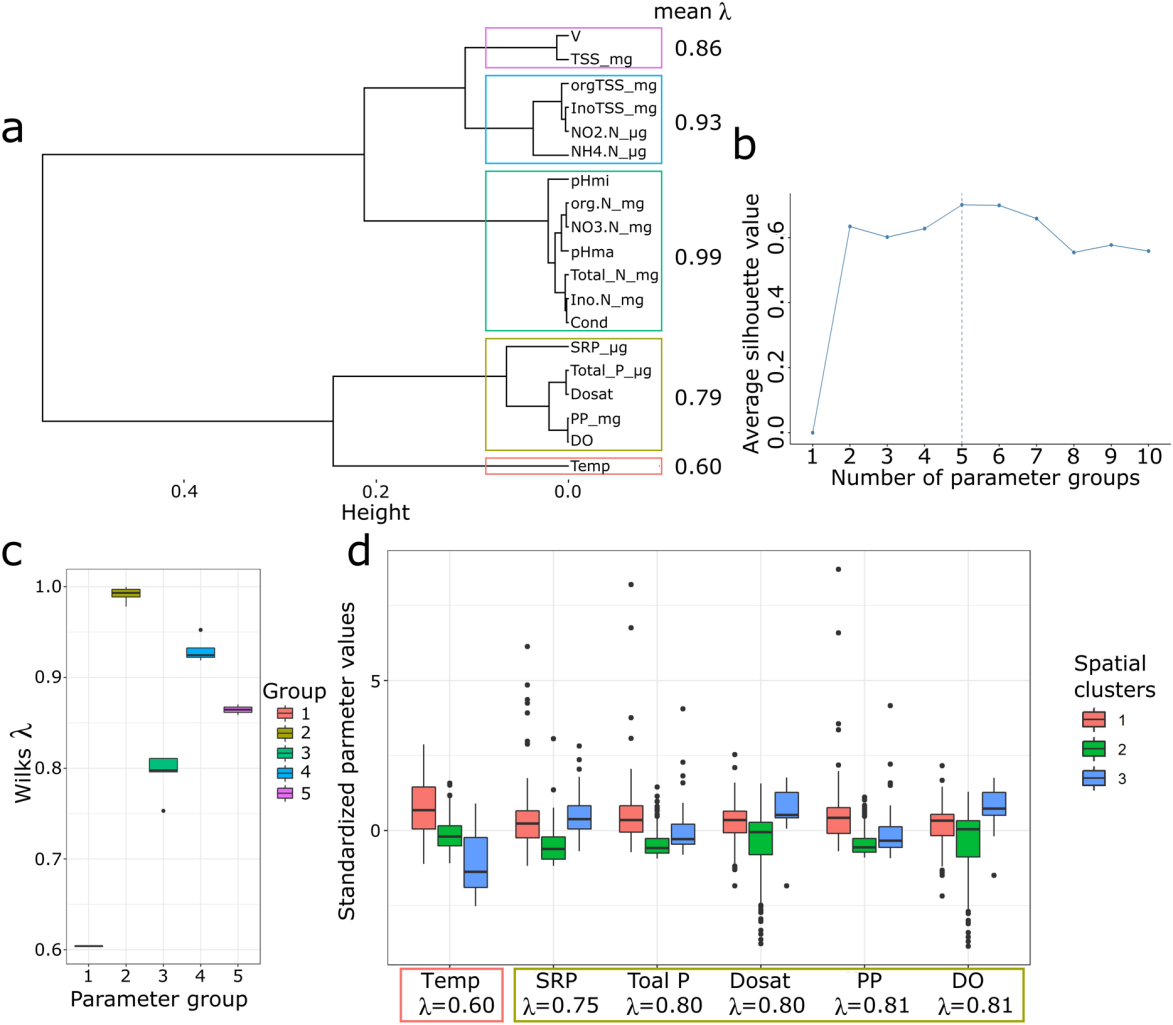
Results of the analysis of Wilk’s *λ* for identifying drivers of spatial variability. **a** The dendrogram showing hierarchical clustering results of all Wilk’s *λ* values. **b** The average silhouette width for each number of clusters, with the selected number of clusters shown with a dashed line. **c** Boxplots of Wilk’s *λ* values for each Wilk’s *λ* group (cluster). **d** Boxplots of standardized values of water quality parameters in the two most important Wilk’s *λ* groups. The colored rectangles show the parameter groups for each parameter

To further analyze the driving factors that lead to these spatial groups, Wilk’s *λ* quotient was estimated for all parameters and clustered in groups. The results of this analysis are shown in Fig. 6a–d. Figure 6a shows the dendrogram of Wilks’ lambda. This dendrogram represents the hierarchical clustering based on Wilks’ lambda (*λ*) values across different WQ parameters. Each leaf on the dendrogram corresponds to a parameter, and the ‘Height’ axis indicates the level of similarity between parameters, i.e., lower heights suggest more similarity. This means that the parameters are grouped based on the similarity in their impact on the differentiation of groups, with the colored lines indicating groupings at various levels of similarity. The mean *λ* values at the top right indicate the average discriminatory power of each cluster of parameters. Figure 6b shows the average silhouette width. The silhouette width measures the cohesion of the WQ parameters within clusters compared to separation from other clusters, with higher values indicating more distinct clustering of the parameters. Part b continues to show how the average silhouette width varies with the number of parameter groups, and the peak (dashed line) indicates our selected number of clusters (5) that optimize within-group similarity and between-group dissimilarity between the different numbers of parameter groups and the chosen optimal number of groups (dashed line). Figure 6c shows boxplots of the distribution of *λ* values for all resulting parameter groups from the dendrogram analysis. High *λ* values suggest a group of parameters with less discriminatory power, whereas lower values suggest a group with more discriminatory power to differentiate between spatial clusters of WQ. Figure 6d shows the boxplots of standardized parameter values of the parameters for the selected WQ parameters within the two most important groups with the lowest *λ* values as determined in Fig. 6c (groups 1 and 3). The distribution of each parameter across the three spatial clusters is visible, allowing for the comparison of central tendency and variability within and between clusters. The chosen clustering based on silhouette width, according to Fig. 6b, aligns with significant differences in *λ* values across groups (Fig. 6c), and therefore, the subsequent boxplots in Fig. 6d confirm these parameters’ varying distributions across spatial clusters. Of these derived five parameter groups, only two show *λ* values < 0.85, hence determining most of the differences between the clusters. These clusters are formed based on similar physico-chemical characteristics, which may reflect underlying spatial patterns within the SMMRB. The WQ parameters like WT, SRP, TP, %Sat, and DO have been identified as significant in distinguishing between the spatial clusters of sites within the SMMRB. The most influential group consists solely of the parameter temperature with *λ* = 0.60, while the second group consists of the parameters SRP, TP, %Sat, and DO (Fig. 6). Parameters in the second group are mainly indicators of anthropogenic influences within the catchment. In this case, *λ* values have helped in identifying which groups of parameters are most important in discriminating between the sites within the SMMRB. The TP and SRP have clearly become significant drivers of spatial variability in water quality within the SMMRB. These findings highlight the importance of phosphorus management in the basin, particularly addressing potential sources, such as untreated wastewater and sediment erosion due to agricultural runoff. While fertilizer application rates were not directly collected in this study, the spatial patterns of elevated TP and SRP in agricultural sub-catchments suggest a mobilization of soil-bound and soluble phosphorus due to management practices like tillage and removal of protective vegetative cover. Mechanical disturbance of soil during field preparation disrupts soil structure, increasing potential for erosion and facilitating the transport of particulate and dissolved phosphorus by water flow paths. These observations show the need for promoting soil and water conservation practices conservation practices in the basin, such as minimum tillage, contour farming (Mwanake et al., 2023).

This approach demonstrates an efficient way to analyze water quality differences between sites using a limited number of key variables. It has allowed pinpointing the key parameters that most effectively can spatially differentiate between the sites with varying surface water quality characteristics. Consequently, it can guide future targeted monitoring and management strategies focusing on mitigating pollution and improving the overall water quality in the SMMRB.

### Seasonal variability in water quality of the SMMRB

The influence of the three seasons was analyzed using Wilk’s *λ* quotient to find parameters that vary greatly in these seasons.

**Fig. 7:**
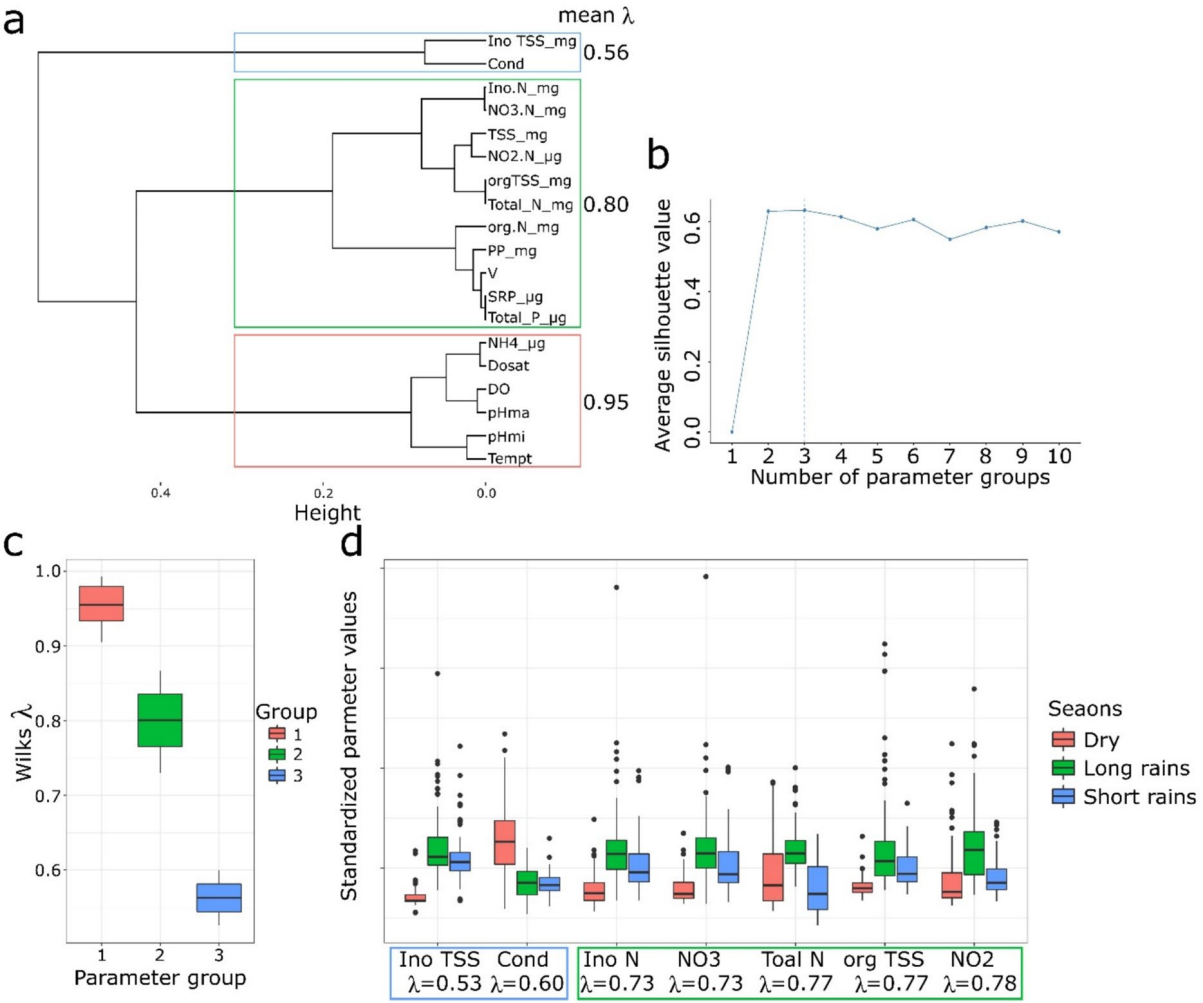
Results of the analysis of Wilk’s *λ* for identifying drivers of seasonal variability. **a** The dendrogram showing hierarchical clustering results of all Wilk’s *λ* values. **b** The average silhouette width for each number of clusters, with the selected number of clusters shown with a dashed line. **c** Boxplots of Wilk’s *λ* values for each Wilk’s *λ* group (cluster). **d** Boxplots of standardized values of water quality parameters in the two most important Wilk’s *λ* groups. For readability, only boxplots of parameters with Wilk’s *λ* values < 0.8 are shown in group 2. The colored rectangles show the parameter groups for each parameter

The results of this analysis are shown in Fig. 7a–d. Figure 7a shows the dendrogram of Wilks’ Lambda which shows hierarchical clustering of WQ parameters based on *λ* value. The ‘Height’ axis indicates how distinct the clusters are; the closer to zero, the more similar the parameters. The colored lines connect parameters into groups based on their similarity in discriminating between the groups as defined by the seasons within the SMMRB. The mean *λ* at the top indicates the average discriminating power of the parameters within each colored group in this case the seasons. Figure 7b shows the average silhouette width, which evaluates the validity of the number of clusters chosen and is a measure of how similar an object is to its own cluster compared to other clusters. The optimal number of parameter groups is identified by the peak in the silhouette width (indicated by the dashed vertical line) in our case three groups. Figure 7c shows boxplots of *λ* values within the three parameter groups identified in Fig. 7a. *λ* values close to 1 suggest that the parameter group has less discriminative power, whereas values closer to 0 suggest greater discriminative power. Figure 7d shows the boxplots standardized of the WQ parameter within the most discriminative parameter groups identified in Fig. 7a, which are those with *λ* values < 0.8 in this case for the two groups (the short rainy season and the long rainy season). The red/dry season group had the highest *λ* values, indicating that the parameters in this group were the least effective in differentiating between the seasons. The parameters that contribute most significantly to differences between seasons include TSS and Cond in the short rainy season, which can explain the dilution effect of increased runoff, leading to lower concentrations of dissolved substances as they are carried away by the influx of rainwater. This dilution effect is typically accompanied by an increase in the TSS as soil particles and other materials are eroded and transported into the river system, while at the same time the conductivity (Cond) decreases due to the reduced concentration of dissolved ions in the larger volume of water. The next category of parameters that also contributes significantly to the differences between seasons belongs to the long rainy season and includes NO_3_^−^ − N, TN, and NO_2_^−^ − N. Comparing values across seasons within the sub-catchments can identify which parameters are most affected by seasonal changes to provide insights into dilution or accretion processes affecting WQ.

The spatial and seasonal variability observed in key parameters, such as TP, SRP, TSS, and nitrogen species, can be directly linked to the land use patterns and the geomorphological characteristics of the SMMRB. The elevated phosphorus concentrations and sediment loads in the Sio and Malaba subcatchments are consistent with intensive agriculture, inadequate soil conservation practices, and steeper terrain (Kitaka et al., 2002), all of which promote particulate phosphorus transport during rainfall events. Furthermore, the field observations of murky, sediment-laden water (see Fig. 3) support this inference. In contrast, the lower nutrient concentrations recorded in the Malakisi sub-catchment reflect less intensive land use and better ground cover, which reduce sediment and nutrient loads. Additionally, the seasonal peaks in nutrient and sediment loads during the rainy seasons highlight the critical role of storm runoff in mobilizing soil and fertilizer inputs into river systems. Climate variability in the region, as documented by the IPCC (2021), suggests that increasing rainfall intensity and variability may further intensify erosion and nutrient pollution in the future. The results of this study show that it is important to integrate land use management, soil conservation measures, and climate adaptation strategies to protect water quality in tropical transboundary basins like the SMMRB.

### Key drivers of water quality within the SMMRB and the potential “hotspots” of water quality parameters

Explorative factor analysis (EFA) was applied to understand the background of the hydrogeochemical, ecological, or anthropogenic processes that determined the water quality clustering patterns at the SMMRB. A detailed explanation of the application of EFA is provided in Appendix B. As shown in Table 6, EFA was applied to all three subcatchments separately to identify which parameters are driving the overall variance of water quality parameters. The resulting overall Kaiser–Meyer–Olkin (KMO) values for the catchments are $KM{O}_{\text{Malaba}}=0.53$, $KM{O}_{\text{Malakisi}}=0.60$, $KM{O}_{\text{Malaba}}=0.64$, indicating that the data is suitable for EFA. Furthermore, Bartlett’s test of sphericity (BST) is highly significant ($p-\text{value}<1{0}^{-16}$) in all catchments. Before applying the EFA, parameters that were highly correlated (> 0.8) with other parameters were excluded from the analysis to avoid redundancy.

**Table 6:** Loadings of the significant factors (with varimax rotation) for the data of the three study catchments

Variables	Factor 1	Factor 2	Factor 3	Factor 4
Malaba sites (four significant factors)				
WT	− 0.06	0.07	− 0.71	0.05
Cond	− 0.15	− 0.09	− 0.55	0.13
pHmin	− 0.05	− 0.03	0.29	0.07
DO	− 0.27	0.10	0.18	− 0.17
TP	**0.93**	0.00	0.14	0.02
SRP	0.69	0.12	0.19	0.04
TN	0.26	**0.91**	− 0.08	0.31
NH_4_^+^ − N	0.36	0.10	− 0.03	0.18
NO_3_^−^ − N	0.37	0.00	0.12	**0.87**
NO_2_^−^ − N	0.73	0.07	− 0.33	0.35
TSS	**0.81**	− 0.05	0.22	0.10
V	0.08	− 0.18	0.74	0.08
Eigenvalues	2.99	1.81	1.72	1.15
% total variance	0.23	0.14	0.13	0.09
Cumulative % variance	0.23	0.37	0.50	0.59
Malakisi sites (three significant factors)				
WT	0.13	0.01	0.27	
Cond	− 0.33	0.03	0.02	
pHmin	− 0.05	− 0.08	− 0.31	
DO	− 0.12	0.28	0.01	
TP	0.74	− 0.26	0.45	
SRP	**0.79**	− 0.12	0.41	
TN	**0.80**	0.52	− 0.30	
NH_4_^+^ − N	0.64	− 0.15	0.20	
NO_3_^−^ − N	0.57	− 0.36	− 0.44	
NO_2_^−^ − N	**0.81**	− 0.16	0.40	
TSS	**0.85**	− 0.12	0.14	
V	0.04	− 0.16	− 0.08	
Eigenvalues	4.09	1.65	1.05	
% total variance	0.31	0.13	0.08	
Cumulative % variance	0.31	0.44	0.52	
Sio sites (three significant factors)				
WT	− 0.15	− 0.07	− 0.21	
Cond	− 0.49	− 0.30	− 0.16	
pHmin	0.00	0.07	0.03	
DO	0.49	0.03	− 0.03	
TP	0.41	0.69	0.22	
SRP	0.48	**0.78**	0.09	
TN	− 0.07	0.22	**0.97**	
NH_4_^+^ − N	− 0.10	0.41	− 0.03	
NO_3_^−^ − N	**0.95**	0.12	0.28	
NO_2_^−^ − N	0.47	**0.80**	0.08	
TSS	0.47	0.59	0.25	
V	0.38	0.07	− 0.08	
Eigenvalues	2.90	2.41	1.65	
% total variance	0.22	0.19	0.13	
Cumulative % variance	0.22	0.41	0.54	

The factor loadings, eigenvalues, and total and cumulative variance are presented in Table 6. It shows various parameters, factor loadings, eigenvalues, and total and cumulative variance. Significant varifactors (VFs) with eigenvalues greater than 1 explain substantial portions of the variance in water quality data. In the Malaba subcatchment, four VFs, having eigenvalues > 1, explained a cumulative variance of 59%.

Bold values indicate strong factor loading (> 0.75). Eigenvalues, percent of total variance, and the cumulative percentage of total variance are given below the loading values for each factor.

The first VF (varifactor) explained 23% of the variance, possessing strong positive varifactor component loadings mainly from TP and TSS. This factor indicates the erosion from the upper sub-catchments, particularly Sio and Malakisi, during rainfall events. This also suggests that the phosphorus in the Malaba sub-catchment has been transported bound to the TSS, likely due to soil erosion processes. Such erosion may be driven by land use alterations, including intensive tillage, reduced vegetation cover, and lack of soil and water conservation practices (Mwanake et al., 2023), which enhance the detachment and mobilization of phosphorus-rich topsoil during rainfall events. Moderate positive varifactor component loadings from SRP and NO_2_^−^ − N were shown, which signifies the influence of dissolved inorganic phosphorus amounts transported. The NO_2_^−^ − N loads are very low and, therefore, are not of concern. The second VF explained 14% of the variance, with TN showing a strong positive varifactor component loading. However, despite its contribution to the variance, the overall levels of TN measured in the basin were not as elevated as initially anticipated, suggesting that TN is not a primary driver of water quality degradation in the SMMRB. This contrasts with more significant indicators, such as phosphorus-related parameters (TP and SRP), which showed stronger correlations with observed water quality measurements. The third VF explained 13% of the variance, with moderate positive varifactor component loadings mainly from velocity and a negative moderate varifactor component loading from WT. This signifies the seasonal impact of V, with higher velocities resulting in cooler temperatures from increased aeration. The fourth VF explained 9% of the variance, with a strong positive varifactor component loading from NO_3_^−^ − N; however, these concentrations were low and did not pose any water quality problems.

In the Malakisi catchment, three VFs, having eigenvalues > 1, explained a cumulative variance of 52%. The first VF explained 31% of the variance, possessing a strong positive varifactor component loadings mainly from SRP, TN, NO_2_^−^ − N, and TSS, indicating the presence of anthropogenic activities and sediment transport. Moderate positive varifactor component loadings from TP, NH_4_^+^ − N, and NO_3_^−^ − N show human influence and signify seasonal changes. The second VF explained 13% of the variance, possessing moderate varifactor component loadings from TN. The third VF explained 8% of the variance, possessing weak positive varifactor component loadings from Total P, SRP, NO_3_^−^ − N, and NO_2_^−^ − N.

In the Sio catchment, three VFs, having eigenvalues > 1, explained a cumulative variance of 54%. The first VF explained 22% of the variance, possessing a strong positive varifactor component. It showed weak negative varifactor component loadings from conductivity. Also noted were weak positive varifactor component loadings from DO, TP, SRP, NO2 − N, TSS, and V, further reflecting anthropogenic influences, sediment transport, and seasonal changes. The second VF explained 19% of the variance, possessing a strong positive varifactor component loadings from NO_2_^−^ − N and SRP. Moderate positive varifactor component loadings from TP and TSS, which signify transport of sediments. The second VF also showed weak positive and weak negative varifactor component loadings from NH_4_^−^ − N and conductivity. The third VF explained 13% of the variance, possessing strong positive varifactor component loadings from TN.

Although financial and time constraints prevented the inclusion of heavy metals and biological parameters, our chosen focus on the water quality parameters adds to and builds upon prior research conducted by Ondoo et al. (2019) and Tenge et al. (2015). Our research has demonstrated the practical implications of the EFA results by identifying key chemical parameters indicative of various environmental processes. Specifically, we have identified parameters associated with erosion and agricultural activity, such as TP, TSS, and SRP. A strong correlation between TP and TSS has been observed in the factor analysis and has shown the significant role of both parameters in the transport and fate of phosphorus within the SMMRB. During rainfall events, particularly in the short and long rainy seasons, increased runoff leads to the erosion of soil particles, as also shown by Kitaka et al. (2002), which are transported downstream as TSS. Particulate phosphorus can bind to these soil particles, resulting in elevated TP levels in surface water (Sharpley et al., 2015). Implementing soil and water conservation measures, for example, covering crops and buffer strips, and using sustainable agricultural practices, including minimal tillage, can help prevent further soil erosion within the SMMRB. H. Mwanake et al. (2023) reported that 60 and 92% of farmers within the basin stated that soil erosion and a loss of soil fertility, respectively, are experienced on their farms. Regular monitoring of TSS and TP is needed to assess the effectiveness of any best management practice interventions and to ensure water quality protection in the basin.

These findings provide a targeted approach for installing surface water monitoring stations and assist with future environmental monitoring and management within the SMMRB, focusing on influential parameters measured in surface waters. This methodology offers an objective basis for determining the spatial distribution of water quality gauging stations in other basins to meet water quality targets.

## Limitations of the study

While this study provides valuable baseline data on water quality dynamics in the SMMRB, several limitations should be acknowledged. First, the temporal resolution was limited to discrete sampling events across three seasons, which may not fully capture short-term fluctuations or episodic pollution events, particularly during storm flows. Second, although land use and geomorphological drivers are discussed in relation to water quality patterns, the study did not directly incorporate quantitative land use mapping or erosion measurements. Future studies integrating remote sensing, high-resolution land use data, and water quality monitoring would strengthen the understanding of pollutant sources. Additionally, this study focused on key nutrient and sediment parameters, but other potential pollutants such as pesticides, heavy metals, or microbiological contaminants were not assessed. Despite these limitations, this work represents a critical step toward understanding the water quality dynamics of a transboundary tropical river basin and provides a solid foundation for future research and management efforts at the SMMRB.

## Conclusion

Our study provides the first comprehensive assessment of spatial and seasonal nutrient dynamics in the transboundary SMMRB. Through a year-long surface water quality monitoring campaign across 12 sites and three distinct seasons, we measured fluctuations in key parameters including water temperature (WT), discharge (Q), pH, conductivity (Cond), dissolved oxygen (DO), total suspended solids (TSS), and nutrient concentrations. Notably, Total Phosphorus (TP) and Soluble Reactive Phosphorus (SRP) concentrations were significantly higher than those typically found in unpolluted rivers worldwide, underscoring phosphorus as a major water quality concern in the basin.

Agglomerative hierarchical clustering effectively grouped the sampling sites into three distinct sub-catchment clusters (Sio, Malaba, and Malakisi), each characterized by unique water quality profiles. The Sio sub-catchment exhibited elevated levels of TP, SRP, TSS, and higher flow velocity, likely driven by intensive agricultural activity and soil erosion. The Malaba sub-catchment showed significant TSS and phosphorus loading transported from upstream areas, while the Malakisi sub-catchment displayed comparatively lower nutrient concentrations due to its headwater location. Seasonal analysis highlighted significant variability, with TSS and conductivity affected during rainy periods, indicating increased sediment transport and dilution effects.

Our analysis of key parameters influencing spatiotemporal variations revealed that elevated TSS and TP concentrations are the main water quality challenges. Exploratory factor analysis (EFA) further identified hydrogeomorphological and anthropogenic drivers of nutrient transport, demonstrating the complex interplay between land use, geomorphology, and hydrology in shaping surface water quality. This integrated statistical approach applied in a tropical, transboundary, data-scarce basin represents a novel contribution that provides transferable insights for other catchments facing similar environmental pressures.

Specifically, for the SMMRB, this study identified regional water quality hotspots and highlighted the significant role of phosphorus in water quality, influenced by land use practices, geomorphological conditions, and seasonal hydrological processes, which are likely to intensify under projected climate variability. A targeted monitoring and mitigation approach is therefore critical, with regular monitoring of TSS, TP, and SRP enabling early detection and response to pollution events. Effective point and non-point source control measures (e.g., soil and water conservation measures, and improved wastewater treatment) are essential for controlling phosphorus loading.

Finally, this study demonstrates that multivariate statistical techniques are effective tools for identifying pollution hotspots and key drivers in tropical river systems, providing a scientific foundation for evidence-based water quality management. Our findings offer actionable guidance for policymakers and resource managers and contribute to the broader conservation and sustainable use of transboundary water resources. Future research in the SMMRB should focus on (1) the long-term impacts of sediment–phosphorus interactions on water quality; (2) the effectiveness of conservation measures at the catchment scale; and (3) continuous monitoring of seasonal dynamics to guide adaptive management. Addressing these gaps will help develop comprehensive strategies for maintaining and improving water quality in the SMMRB and similar tropical river basins globally.

## Figures and Tables

**Fig. 8:**
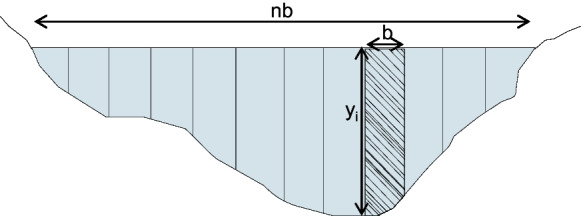
Stream cross section

## Data Availability

The authors declare that the data supporting the findings of this study are available within the manuscript, any additional data can be requested via email.

## Appendix

### Appendix A1: Sample handling and laboratory analysis

GPS technology accurately identified sampling sites. Samples were collected, preserved, and transported following APHA, (2012) standards to ensure the reliability of water quality analysis. The study encompassed the following key steps:

- Sampling: Sampling points were systematically and representatively sampled using clean, acid-prewashed bottles to prevent contamination and ensure precise results.
- Preservation: Ice packs in compact cooler boxes were used to preserve water samples in the field, maintaining sample integrity and chemical stability during transportation to the laboratory.
- Transportation: Samples were promptly transported to the laboratory after the final site was sampled to minimize potential changes or degradation. Additional ice packs were added to cooler boxes before the return trip, and samples were carefully checked to ensure proper sealing, preventing spills or leaks that could compromise sample quality or contaminate other samples.
- Record Keeping: Detailed documentation of the sampling process, including sample location, date, time, sample identifier, and relevant field observations such as visible human activities or direct pollutant sources, was maintained to ensure traceability and quality control. These records facilitated accurate sample identification and data association during laboratory analysis.

Upon arrival at the laboratory, the samples underwent filtration using glass-fiber filters (Whatman GF filters). A portion of each water sample remained unfiltered, as the analysis of Total Nitrogen and Total Phosphorus required unfiltered samples. Total Nitrogen (TN) analysis employed the persulphate method (Koroleff, 1983), which oxidizes all nitrogenous compounds to nitrate. Total Phosphorus (TP) determination involved digesting and reducing phosphorus forms into free orthophosphate form (SRP) using persulphate digestion, followed by ascorbic acid method analysis (APHA, 2012) on filtered samples. The widely used method for analysing soluble reactive phosphorus in natural waters, reported by Murphy and Riley, (1962), was employed (Worsfold et al., 2016). Total Suspended Solids (TSS) were estimated gravimetrically on glass-fibre filters (Whatman GF/C filters) after drying at 95 °C. Soluble Reactive Phosphorus (SRP) was determined using the ascorbic acid method on filtered samples. Nitrate nitrogen (NO_3_^−^-N) was determined using the sodium-salicylate method (APHA, 2012; Radecki & Wesołowski, 1976), while Nitrite nitrogen (NO_2_^−^-N) was assessed via the Griess-Saltzman method, which forms a reddish-purple azo dye at pH 2.0–2.5. Ammonium nitrogen (NH_4_^−^-N) was determined using the hypochlorite method with nitroprusside as a catalyst. Adhering to standard methods, such as those outlined by the APHA, (2012), ensures consistency in water quality analysis across laboratories and facilitates result comparison. Following these protocols minimizes errors, maintains sample integrity, and enhances the reliability and accuracy of water quality assessments.

### Appendix A2: Statistical Analysis

#### Agglomerative Hierarchical Clustering

Agglomerative Hierarchical clustering (Ward, 1963) represents a clustering method that relies on distances between data points. Unlike other clustering algorithms that aim to segregate the data into a predetermined number of clusters, hierarchical clustering constructs a hierarchical structure comprising clusters of varying granularities (Cai et al., 2014).

In agglomerative hierarchical clustering, each observation initially forms its cluster and then merges into larger clusters based on a distance metric and a linkage criterion. The linkage criterion determines how the distance between a set of observations is computed using their pairwise distances. This study adopts the Euclidean distance and Ward’s criterion (Ward, 1963).

The graphical representation of the clustering outcomes is depicted by a dendrogram, illustrating groupings based on the distance between clusters. The optimal number of clusters is determined by scrutinizing the dendrogram and calculating the silhouette value (Rousseeuw, 1987) for each cluster count.

The silhouette value assesses the similarity of a data point to its assigned cluster relative to other clusters.

#### Wilk’s lambda quotient distribution

Wilk’s *λ* quotient (Wilks, 1932) is employed to ascertain the influence of each water quality parameter on the various groups and is commonly utilized in discriminant analysis. Mathematically it is defined as

$$\lambda = \frac{\sum_i\sum_j\left(x_i -\bar{xi}\right)^{2}}{\sum_i\sum_j\left(x_i -\bar{xi}\right)^{2}}$$

where 𝑥𝑖𝑗 denotes a data point j of cluster i with a cluster mean 𝑥𝑖 and 𝑥 the total mean. It is the ratio of the within-group sum of squares to the total sum of squares. Wilk’s *λ* can assume values between 0 and 1, where 0 signifies total discrimination and 1 implies no discrimination. Therefore, values closer to 0 indicate distinct means and, consequently, influence group formation.

#### Explorative factor analysis (EFA)

Exploratory Factor Analysis (EFA) endeavours to identify a specific number of latent factors that elucidate the observed data. In the context of the SMMRB, various activities and actions could potentially explain or contribute to the status of water quality. These may encompass erosion from agricultural fields, runoff into the rivers during the rainy season, and anthropogenic activities such as farming on buffer zones, which can result in the loss of topsoil and subsequent sediment transport downstream, among other factors. EFA aims to uncover and characterize these underlying factors to better understand the complex dynamics influencing water quality in the SMMRB. EFA can be expressed as:

$$z_{j}\begin{array}{cc}=a_{j1}F_{1}+a_{j2}F_{2}+\dots +a_{jm}F_{m}+e_{j}, & with j=1, 2, \dots p\end{array}$$

where p denotes the total number of variables, 𝑧𝑗a single variable, F_*1,2,...m*_the underlying factors and e_j_ the residual term accounting for errors or other sources of variation.

Before applying Exploratory Factor Analysis (EFA), the suitability of the dataset for factor analysis is assessed using two key tests: the Kaiser–Meyer–Olkin (KMO) test (Kaiser, 1974) and Bartlett’s sphericity test (BST) (Bartlett, 1954). The KMO test evaluates whether a given dataset can be adequately described by underlying factors and estimates the proportion of variance among variables that might be common variance. KMO values range between 0 and 1, with higher values indicating greater suitability for factor analysis. (Kaiser, (1974) suggests a minimum KMO value of 0.5. In this study, the KMO value obtained was 0.81, indicating excellent suitability. On the other hand, Bartlett’s sphericity test (BST) assesses whether the correlation matrix significantly differs from a diagonal matrix, wherein variables would be unrelated. A significant result indicates that the variables are interrelated and thus suitable for factor analysis.

The number of factors is estimated through parallel analysis (Horn, 1965), a method that compares the ordered eigenvalues of the correlation matrix with simulated eigenvalues generated from randomly sampled correlation matrices. When the eigenvalue of a factor exceeds the corresponding simulated eigenvalue, it suggests valuable information within that factor, as eigenvalues represent the variance accounted for by a factor.

Factor Analysis is conducted using the Maximum Likelihood method, deemed most appropriate in cases where residual variance reflects normally distributed errors (Fabrigar et al., 1999; Revelle, 2009). Subsequently, the resulting factors undergo varimax rotation (Kaiser, 1958) to generate orthogonal factors with a simplified structure, thereby enhancing interpretability (Revelle, 2009).

For each of the sub-catchments within the SMMRB, factor loadings corresponding to a number of significant varifactor components (VFs) is expected. These loadings represent the correlation between the variables and the potential VFs. The loadings range between -1 and 1, with values closer to -1 or 1 indicating a stronger relationship between the variable and the VFs. Values closer to 0 indicate a weaker relationship. According to Khanday et al., (2021), factor loadings estimated from PCA are considered as strong, moderate and weak with an absolute loading value of > 0.75, between 0.75–0.50 and 0.50–0.30 respectively. For each sub catchment, several VFs can be considered"significant"based on their contribution to the total variance, which indicates how much of the overall variability in the data these VFs explain. Each VF has an associated eigenvalue that indicates the amount of variance in the overall data it explains. Also a VF is considered significant if its eigenvalue is greater than 1, as it contributes more to explaining the variance than a single observed variable.

### Appendix A3: Determination of SMMRB Discharge

For the Sio, Malaba and Malakisi river systems the method that was used to calculate the discharge was the velocity area method following standard hydrological procedures.

## Materials Used

Dip stick, metre rule, tape measure, rotating element flow meter, waders, calculator, stop watch.

## Procedure

### Calculating the velocity of the rivers

i. Using Floaters
  In the sampling points where the stream channels were shallow, discharge was directly calculated using the float method. This was because the rotating element flow meter was too large to make any rotations. Discharge was approximated from simple measurements according to (Davis, 1938) formular Q = wdla/t 
  where Q is discharge in meters per seconds, w is width in m, d is mean depth in m, I is distance in mover which a float travels in time, t, in sec, and a is a coefficient that varies with the nature of the sediment, [0.8 if rough and 0.9 if smooth (mud, sand, bedrock). The coefficients are used in this case because surface velocities are typically higher than mean or average velocities.
  Alternatively one can estimate the surface velocity using a floater by measuring the time taken over a measured distance, velocity can be calculated as; Velocity (M/S) = Distance (M)/time (s) Then use the method explained below to calculate the cross sectional area after which discharge can be computed as: Discharge (M^3^/sec) = Area (M^2^) * Velocity (M/sec),
ii. Using the rotating element flow meter
  The velocity was calculated using the rotating element flow meter at each depth as indicated below in Fig. 8. The principal of using the rotating element flow meter is to convert the propeller revolutions into a horizontal distance as shown in the formula below.
  - Distance (M) = Counts (Final – initial) * Rotar Constant (26,873))/999999 (manufacturers constants)
  - Velocity/Speed (M^-1^ s) = (Distance (M)/time (s)

### Calculating the area of the river channel

The river channel width is divide into the **n** sub-sections (see figure) with an equidistance width of **b** meters. The number of the subsections given by **n** in the figure below should depend on the size of the river. A measuring tape was stretched across the river cross-section to define the width at each site (Fig. 8).

The water depth **y** is determined at the centre or beginning of each cross section using a dipstick or meter rule.The velocity is determined at 0.6 depths from the top or 0.4 depth from the bottom (which is the average velocity v_i_) at intervals of say 0.5 m or 1 m across the channel. (depending on the width of the entire river channel).

The following parameters were then calculated.

- Cross sectional area of each sub-section.
- Mean water velocity across the channel
- Waters discharge Q (m^3^/s) as: $Q=\sum_{i-1}^{n}{v}_{i}{A}_{i}$

Where v*i* is the mean velocity (m/s) and A is the cross sectional area (m^2^).

Discharge was then calculated as;

i) Discharge (M^3^/sec) = Area (M^2^) * Velocity (M/sec), where
ii) River Cross section area (M^2^) = as area under the curve by plotting water depths across the channel. This corresponds to the wetted cross-sectional area of the river at the time of sampling, calculated based on observed depth and width at each site and season. These values naturally vary across seasons due to changes in water levels. Rather than the full channel morphology.
iii) Velocity (as obtained in part a roman i or ii above

Malaba Station day 1 of sampling: Discharge 0.4m^3^/sec.

Malaba Station day 3 of sampling: Discharge 0.30m^3^/sec.

Malaba Station day 4 of sampling: Discharge 0.27m^3^/sec an automatic propeller was used.

Malaba Station day 5 of sampling: Discharge 0.5m^3^/sec.

Malaba Station day 5 of sampling: Discharge 0.5m^3^/sec This day there was heavy rain for like half an hour.
